# Multiplexed, High Density Electrophysiology with Nanofabricated Neural Probes

**DOI:** 10.1371/journal.pone.0026204

**Published:** 2011-10-12

**Authors:** Jiangang Du, Timothy J. Blanche, Reid R. Harrison, Henry A. Lester, Sotiris C. Masmanidis

**Affiliations:** 1 Division of Biology, California Institute of Technology, Pasadena, California, United States of America; 2 Kavli Nanoscience Institute, California Institute of Technology, Pasadena, California, United States of America; 3 Broad Fellows Program in Brain Circuitry, California Institute of Technology, Pasadena, California, United States of America; 4 Redwood Center for Theoretical Neuroscience, Helen Wills Neuroscience Institute, University of California, Berkeley, California, United States of America; 5 Intan Technologies, Los Angeles, California, United States of America; Max-Planck-Institut für Neurobiologie, Germany

## Abstract

Extracellular electrode arrays can reveal the neuronal network correlates of behavior with single-cell, single-spike, and sub-millisecond resolution. However, implantable electrodes are inherently invasive, and efforts to scale up the number and density of recording sites must compromise on device size in order to connect the electrodes. Here, we report on silicon-based neural probes employing nanofabricated, high-density electrical leads. Furthermore, we address the challenge of reading out multichannel data with an application-specific integrated circuit (ASIC) performing signal amplification, band-pass filtering, and multiplexing functions. We demonstrate high spatial resolution extracellular measurements with a fully integrated, low noise 64-channel system weighing just 330 mg. The on-chip multiplexers make possible recordings with substantially fewer external wires than the number of input channels. By combining nanofabricated probes with ASICs we have implemented a system for performing large-scale, high-density electrophysiology in small, freely behaving animals that is both minimally invasive and highly scalable.

## Introduction

Neural probes comprising multiple extracellular microelectrodes have proven to be an effective tool for recording activity from large neuronal ensembles [1]. In contrast to most imaging methods, implantable probes can access virtually any depth of the brain and, because of their small size, are more conducive to measurements in awake, freely behaving animals. Much progress has been made in neural probe technology over the last 6 decades since the pioneering tungsten microelectrode experiments of Hubel and Wiesel [2], [3]. By scaling up from single to multi-channel electrodes, it is now possible to record spikes from well over 100 neurons [4], with the number of simultaneously recorded single-units doubling roughly every 7 years [5]. However, this apparent wealth of data overlooks an important limitation of existing technology: densely recording multiple units in the same region of the brain in a minimally invasive fashion remains a daunting challenge. This limitation must be overcome in order to decipher how the brain encodes information across various scales, from locally connected microcircuits to long-range correlations between macrocircuits [6]. Instrumentation noise, the rapid spatial decay in extracellular action potential amplitude, and interference from more distant co-active neurons, means that electrodes must lie within ∼100 µm of the soma to reliably detect and isolate a neuron [7]–[9]. There is therefore a strong impetus to develop higher density electrode arrays to faithfully monitor neuronal subpopulations within discrete anatomical regions.

Microelectromechanical systems (MEMS) based electrode arrays are increasingly being used to address this challenge [10], [11]. For example, silicon probes containing dozens of recording sites on a thin penetrating shaft have yielded important insights into the function of the hippocampus [12] and visual cortex [13], [14]. However, the development of such devices inevitably involves a tradeoff between the number of recording sites, the device width, and the ability to connect the electrodes. This issue becomes more salient as the number of electrodes per shaft is scaled up, requiring an increase in the width of the device to accommodate additional electrical leads (which are also known as interconnects). Existing neural probes employ ≥1 µm lead width and separation [15], [16]. We used electron-beam (e-beam) lithography to reduce these features to sub-micron dimensions. The resulting high-density lead devices can accommodate a large number of recording sites without an appreciable increase in probe width (Table S1).

Performing in vivo very large-scale electrophysiology with multichannel devices presents an additional challenge: interfacing probes with the external instrumentation to record neural signals. Thus, miniaturizing the instrumentation is another crucial requirement for successfully scaling up neural probe recording capabilities. The combination of implantable neural interfaces with complementary metal-oxide-semiconductor (CMOS) electronics can fulfill this role [17], [18], similarly to what this advance has done for in vitro microelectrode arrays [19], [20]. Using a newly developed ASIC, we demonstrate low noise, wide band, multiplexed measurements with 64 channel nanofabricated neural probes.

## Results

### Nanofabricated silicon-based neural probes

**Figure 1:**
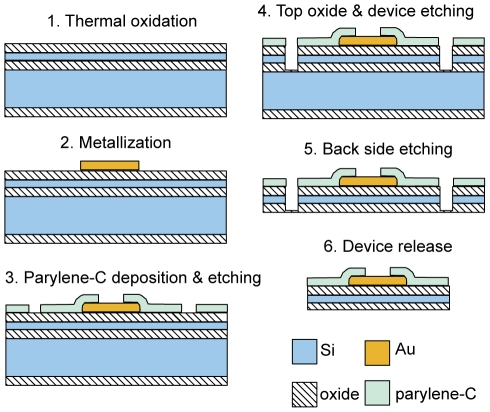
Process flow schematic for the nanofabrication of 64 channel silicon neural probes.

**Figure 2:**
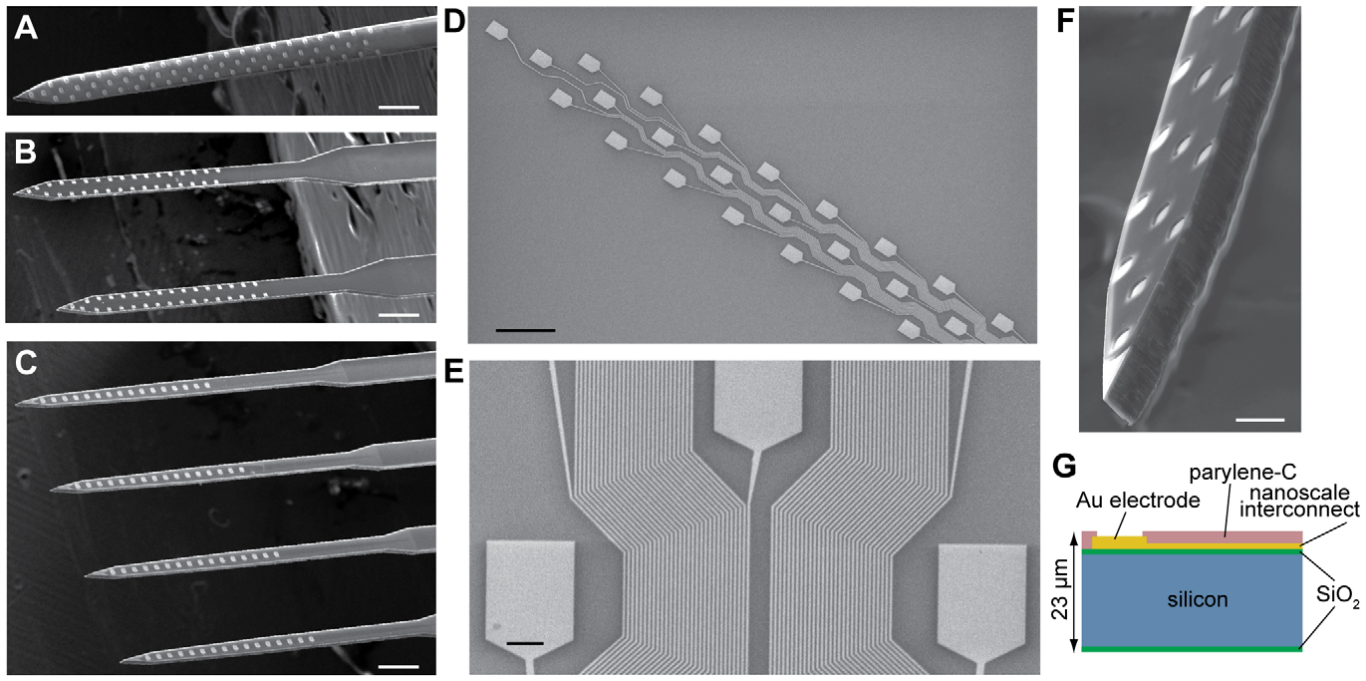
64 channel silicon-based neural probes with nanoscale leads. (**A**–**C**) Three configurations of probes. Scale bars, 200 µm. (**D**) Gold recording sites and nanoscale leads, patterned with e-beam lithography. The lead width ranges from 1000 nm at the top lefthand corner, to 290 nm at the bottom righthand corner of the image. Scale bar, 50 µm. (**E**) A section of interconnecting leads belonging to the “honeycomb” array configuration displayed in (A) and (D). The narrowest traces have width and spacing of 290 nm. Scale bar, 5 µm. (**F**) Characteristic chisel-shaped profile of the probes resulting from deep reactive ion etching of silicon. Scale bar, 25 µm. (**G**) Silicon device cross-section.

Neural probe fabrication was carried out on 100 mm diameter silicon-on-insulator (SOI) substrates (Figure 1). We developed three different 64-channel electrode arrays incorporating various electrode configurations (Figures 2A–C). High-resolution e-beam lithography was utilized to define nanoscale gold leads and 108 µm^2^ electrode recording pads. The e-beam process produced leads with a width and spacing as narrow as 290 nm on certain portions of the array (Figures 2D,E).

**Figure 3:**
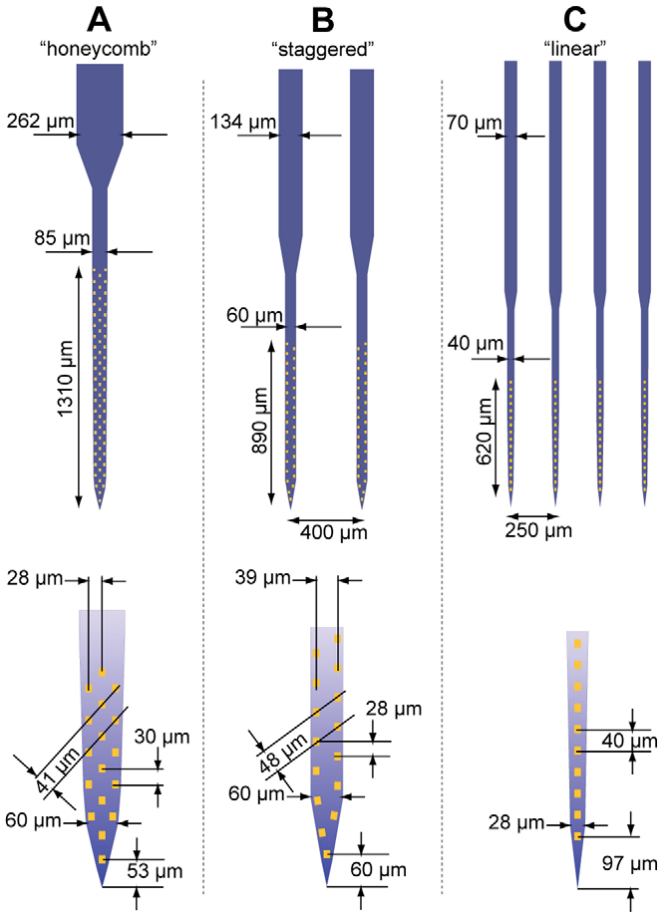
Detailed schematic of the three fabricated probe configurations. All probes contain 64 independent recording channels. Device (**A**) contains 290 nm leads at the most space-limited area of the array on the base of the shaft. The shaft width is tapered from 60 µm at the tip to 85 µm at the base. The 262 µm ‘stalk’ indicates the location at which lead fabrication was transferred from e-beam to a standard UV lithography process with 3 µm feature size. Device (**B**) contains 350 nm leads, 32 sites per shaft, and its shafts are 60 µm wide. Device (**C**) contains 500 nm leads, 16 sites per shaft, and its shafts are tapered from 28 to 40 µm. All array configurations were fabricated in two device length variants (2.5 or 5.5 mm long shafts).

Deep reactive ion etching was used to release the probes from the SOI substrate (Figure 2F) [15], [21]. The total shaft thickness was 23 µm (Figure 2G), and tip widths ranged from 28 µm for the linearly configured array in Figure 2A, to 60 µm for the “honeycomb” array in Figure 2C. These structures were tapered such that the basal shaft widths were 40 and 85 µm, respectively. Detailed array schematics with relevant dimensions are provided in Figure 3.

### Integrated circuitry for signal amplification, filtering, and multiplexing

**Figure 4:**
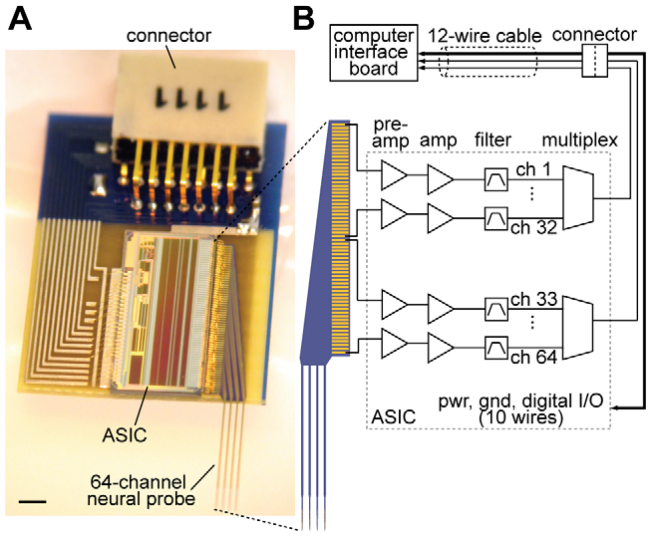
A fully integrated 64 channel recording system. (**A**) The system, weighing 330 mg, is built on a 9 mm×15 mm printed circuit board containing a nanofabricated neural probe, ASIC, and miniature connector. The epoxy encapsulating the ASIC is omitted to show the wire bonds connecting various components on the board. Scale bar, 1 mm. (**B**) Schematic illustrating the signal transduction path. Pre-amplification = 100x; amplification = 2x; filter = 1 to 6,400 Hz; multiplexing ratio = 32∶1. Note that only 6 wires are needed to operate the system in recording mode; the remaining wires are used for impedance, amplifier tests, and electroplating.

An ASIC for performing all essential signal buffering, amplification, filtering, and multiplexing functions was developed using 350 nm CMOS process technology (see Table 1 for key device parameters). The contacts for the 64 input channels were arranged in a single row on one side of the chip, and their spacing was matched to those on the neural probe to facilitate wire bonding on a compact printed circuit board (PCB), displayed in Figure 4A. The assembled PCB, measuring 9 mm×15 mm and weighing 330 mg after epoxy encapsulation, also contains a miniature connector, and surface mount passive components for performing voltage stabilization and division for powering and digitally controlling the ASIC. The placement of the probe and accompanying head stage circuitry on the same PCB was motivated by efforts to minimize the system's size, weight, and noise from external interference. Figure 4B illustrates the operating scheme of a 64 channel neural probe by the ASIC. Signals were amplified, filtered, and sent through two 32∶1 analog multiplexers before being transmitted off the PCB via a connector which provided an electrical interface to a data acquisition (DAQ) device. A minimum of 6 wires (power, ground, channel sweep, channel synchronization, and the two multiplexer outputs) were required to perform recording functions with the ASIC; the remaining connections were required to test electrode impedances and amplifier operation.

**Table 1:** Measured specifications of the application-specific integrated circuit used in these experiments.

Parameter	Value
Number of input channels	64
Number of wires needed for recording	6
Size	8.2 mm×3.5 mm
Power supply voltage	3.0 V
Power consumption	22.5 mW
Low-frequency cutoff	1.3±0.2 Hz
High-frequency cutoff	6,400±300 Hz
Amplifier input referred noise (f = 1-6,500 Hz)	2.0 µV_rms_
Amplifier input referred noise (f = 100–6,500 Hz)	1.7 µV_rms_
Gain	194 V/V
Amplifier input impedance (1 kHz)	13 MΩ
Average common-mode rejection ratio (60 Hz)	83 dB
Average power supply rejection ratio (60 Hz)	84 dB
Average adjacent input channel crosstalk (1 kHz)	−84 dB

Note the reduction in number of wires needed for recording relative to the number of inputs, which is attributed to on-chip multiplexers.

### Low noise characteristics of the recording system

**Figure 5:**
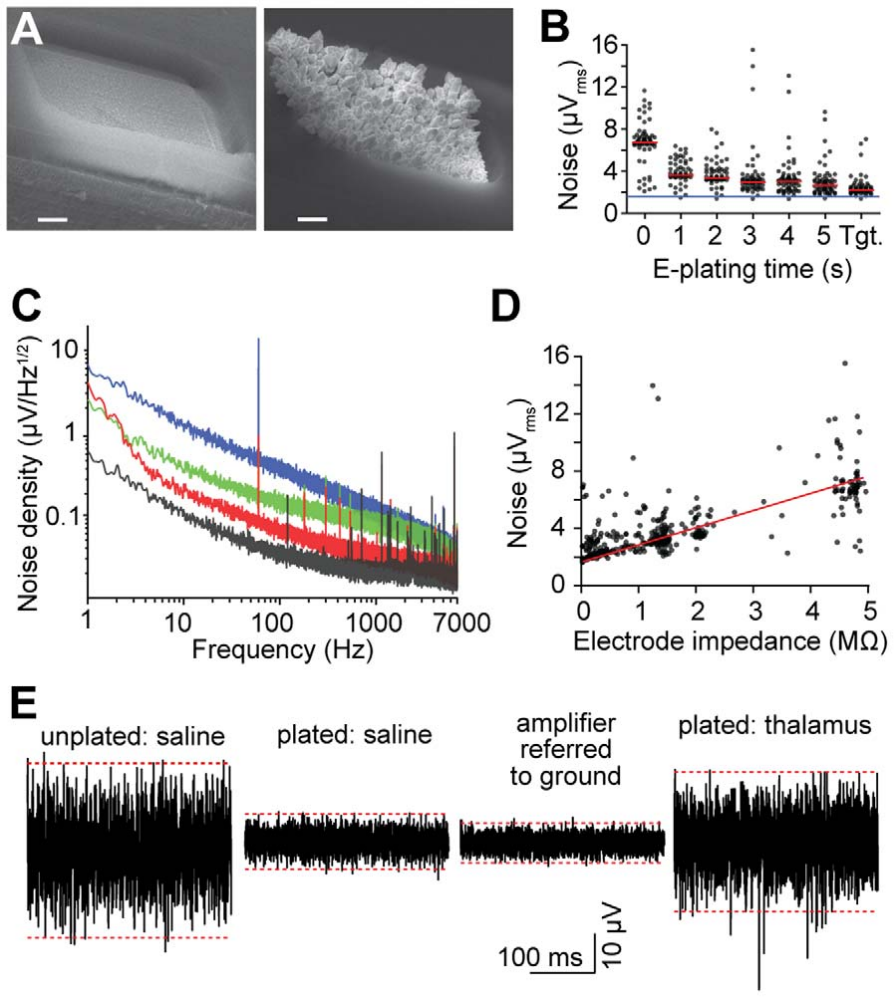
Low noise performance of the multiplexed recording system. (**A**) Scanning electron micrographs of 108 µm^2^ electrodes in their original fabricated state (left), and after gold electroplating to an impedance of 0.15 MΩ. Scale bars, 2 µm. (**B**) Root-mean-square noise of the electrophysiological recording system as a function of electroplating time. Measurements were performed in phosphate buffered saline. Tgt. denotes plating proceeded until a target impedance of 0.4 MΩ per channel was achieved. Signals correspond to the 0.1–6.5 kHz frequency band. Black points denote individual channels; red lines represent median value of 64 sites; the blue line corresponds to the input-referred noise floor of the amplifiers. (**C**) Amplifier noise spectral density of a 0 (black), 0.4 (red), 2.2 (green), and 4.9 MΩ (blue) impedance electrical connection to ground. (**D**) Root-mean-square noise in the 0.1–6.5 kHz band as a function of electrode impedance at 1 kHz. The red line is for illustration purposes. (**E**) Segments of single-site recordings under various electroplating and environmental conditions. Dashed red lines represent the 99.9^th^ percentile range of the voltage. Unplated and plated electrodes have an impedance of 4.9 and 0.3 MΩ, respectively. The thalamic recording was carried out in a mouse under chloral hydrate anesthesia. Note that the noise level for a plated probe in thalamus is lower than that of an unplated probe in saline, in spite of background neural activity.

To investigate the noise performance of the multiplexed recording system, we utilized additional circuitry on the ASIC, which can also perform rapid measurement of electrode impedance, as well as modification of impedance via electrodeposition (Figure 5A). The noise level markedly decreased with consecutive gold electroplating cycles (Figure 5B), and asymptotically approached the intrinsic noise of the amplifier (1.7 µV_rms_, f = 0.1–6.5 kHz). This trend toward lower noise was strongly correlated with the accompanying reduction in site impedance (Figure 5C,D), confirming that gold electroplating is an effective way to minimize noise of electrodes with small geometric areas [22]. Figure 5C also indicates that the level of 60 Hz interference in a relatively unshielded laboratory recording environment (fluorescent ceiling lights turned on; no Faraday cage) dropped significantly post-plating, presumably because of the lower electrode impedance. Although additional interference peaks appear in the spectrum of gold-plated electrodes, they contribute <2% to the total noise power and thus do not significantly disrupt recording quality. Figure 5E depicts demultiplexed and filtered segments from extracellular measurements under different impedance and recording conditions, further illustrating the benefits of electroplating for lowering noise.

### Crosstalk and thermal noise in high-density leads

Impedance measurements between adjacent wires yielded 39±7 MΩ (mean±1 s.d. at 1 kHz). Given that the measured gold-plated electrode impedance values are only ∼1% of this value (see Materials and Methods), we expect a negligible level of capacitive cross-talk within these devices. Furthermore, thermal dissipation in the electrical leads contributes noise to electrophysiological recordings. The RMS noise over a bandwidth of Δf can be calculated by the expression:
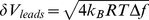
(1)where k_B_ is the Boltzmann constant, R is the resistance of the wire, and T is the temperature. Resistance measurements across the length of the probe containing 290 nm leads yielded 9.2±0.4 kΩ, which corresponds to δV_wire_ = 1.0 µV_rms_ for a bandwidth of 6.4 kHz. This contribution is less than the reported intrinsic noise from the amplifier and electrode-fluid interface; however, the fabrication of significantly longer or narrower leads is likely to produce more noise from thermal dissipation in the wires.

### Multiplexed, large-scale data acquisition

**Figure 6:**
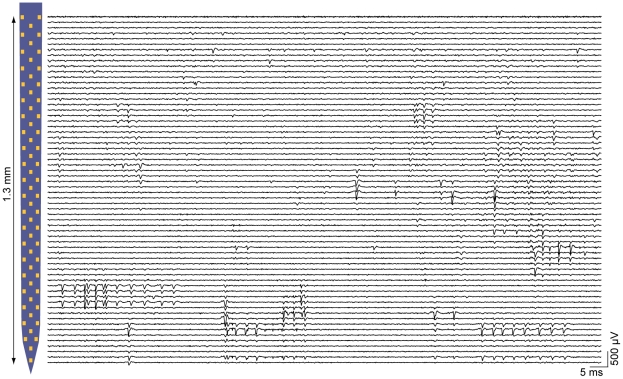
Parallel recording capabilities of the multiplexed electrophysiology system. Traces are filtered from 0.3–5 kHz to highlight spiking activity, and are plotted near their corresponding location on the shaft of the probe displayed on the left (for each horizontal left-right pair of pads, the trace for the left-hand pad appears just above the trace for the right-hand probe). Measurements were made in the mouse thalamus with a probe with post-plating impedances of 0.6±0.4 MΩ (mean±1 s.d.). The majority of sites report action potentials, and only one of the 64 sites (uppermost trace) is non-functional.

**Figure 7:**
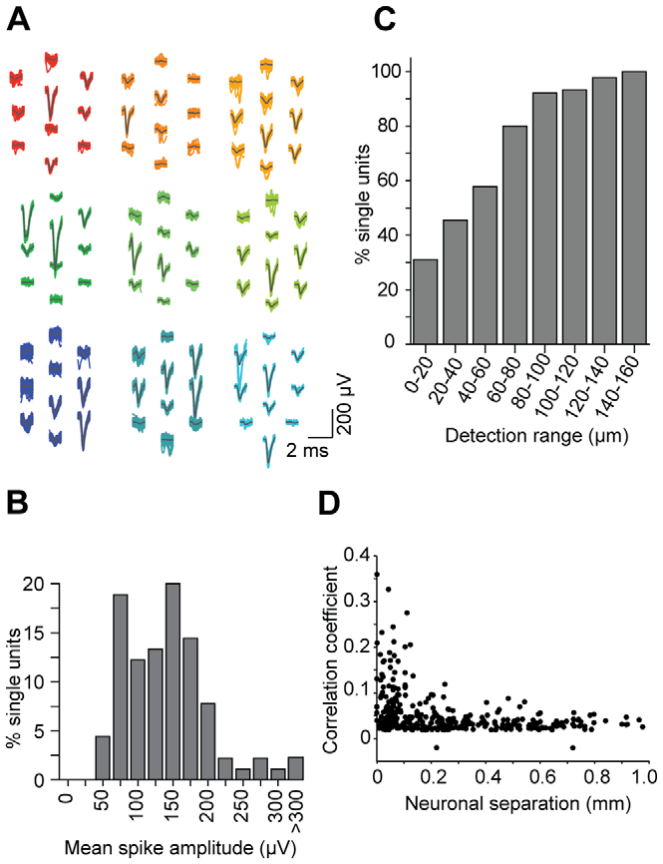
Nanofabricated probes facilitate high-density electrophysiological recordings. (**A**) Representative waveforms corresponding to putative single neurons. Gray lines denote the mean waveform. (**B**) Distribution of the maximum peak-to-peak amplitude per unit. The cutoff for reliable spike detection was 50 µV. (**C**) Cumulative distribution of the maximum distance across which a unit could be detected (defined as a mean spike-triggered waveform >50 µV). 31% of units were observed on only a single site; the remainder were measured on ≥2 recording sites, which have a nearest neighbor spacing of ∼40 µm. (**D**) Spike time cross-correlation coefficient from 54 simultaneously recorded putative thalamic units, as a function of estimated inter-somatic separation.

Having demonstrated the low noise recording characteristics of our system, we next evaluated its ability to record neuronal action potentials. Figure 6 displays a representative epoch of spontaneous firing activity in the ventral posteromedial thalamic nucleus of an anesthetised mouse. The majority of functional (63/64) electrodes in the 1.3 mm long array report spiking activity, much of which appears to be correlated between neighboring sites, indicating that >1 electrode often records from the same neuron. The close electrode spacing is thus well suited for high-density electrophysiology with extensive coverage of extracellular fields along the length of the array. The combination of microvolt level intrinsic amplifier noise and recording site modification via electroplating enabled reliable spike detection with peak amplitudes as low as 40 to 50 µV. Waveforms from a subset of the putative neurons isolated from the recording in Figure 6 are displayed in Figure 7A. The maximum spike amplitude per unit was 150±80 µV (mean±1 s.d; Figure 7B), which is in good agreement with other extracellular recordings with comparable electrode properties [11], [16]. Using a detection threshold of 50 µV in the 0.4–5 kHz band, we found that the spike signature of ≈69% of recorded units could be observed across at least 40 µm (Figure 7C), which is equivalent to the average adjacent site spacing on this particular array. The concurrent measurement of extracellular action potentials on multiple channels is an effective way to improve single-unit analysis [9], [11]. The inter-electrode distance on this array appears to offer such signal redundancy for the majority of measured neurons, but these results suggest the classification of a small subset of cells could benefit from <40 µm site spacing.

One of the principal advantages of simultaneously recording multiple neurons is the ability to assess functional interactions via cross-correlation analysis [23]. This technique may augment the analysis of neural circuit information processing [24], and also provide insights into the synaptic connectivity of local circuits. To investigate the latter, we measured the spike time cross-correlation coefficients for 54 thalamic neurons recorded in parallel with the 1.3 mm long array. We compared the correlation coefficient of each pair of cells to their inter-somatic separation (Figure 7D). The location of the soma was estimated by the mean spike field position on the electrode array, weighted by the peak-to-peak spike amplitude. The figure displays results from 356 of 1,431 total pairs, which exhibited significant correlations (p<0.05) on the timescale of ±20 ms. We observed a pronounced increase in the mean correlation between more proximally located thalamic neurons, qualitatively consistent with the increased short-range connection probability observed for a different group of neurons in cortex [25]. Although neuronal correlations are known to strongly depend on the behavioral state [24], and may therefore be disrupted by anesthesia, this analysis demonstrates the potential of this recording system to link functional and anatomical aspects of neural circuits with high spatial resolution.

### Measurements in freely behaving mice

**Figure 8:**
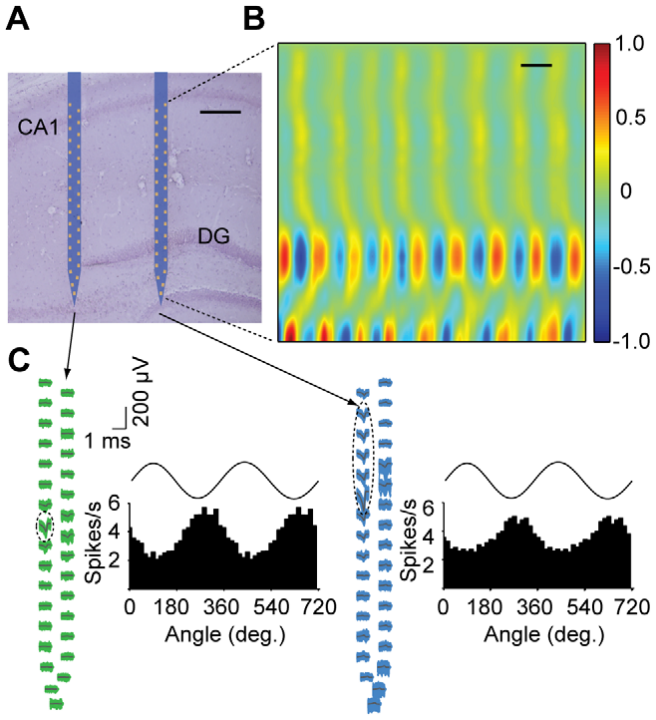
Electrophysiology with nanofabricated probes in awake behaving mice. (**A**) Nissl-stained brain section overlaid with a schematic of the probe at its stereotaxically implanted location. Each silicon shaft is 60 µm wide. Scale bar, 200 µm. (**B**) Current source density analysis of local field potentials across the hippocampus with a vertical resolution of ∼28 µm. Measurements correspond to the right-most shaft, which is co-localized with both the supra- and infra-pyramidal blades of the dentate gyrus (DG). The data were gathered during home cage exploratory behavior. The CSD is normalized to ±1. Scale bar, 100 ms. (**C**) Waveforms of two putative single units recorded from this probe across all sites on their entire respective shafts, with histograms showing theta phase locking of spikes. Dashed ellipses indicate the sites exhibiting the highest extracellular action potentials for these units. Measured theta oscillations on top of the histograms are for reference. Theta oscillations were measured from the upper right-most electrode near the CA1 pyramidal cell layer.

The low form factor and weight, combined with the reduced wiring requirements of our multiplexed recording system, makes it particularly well suited for large-scale electrophysiology in small freely behaving animals. The 330 mg system described here weighs ∼1.5% of an adult mouse, representing a substantial improvement over other systems with comparable numbers of recording sites, when considering the combined weight of the electrode and amplifier assemblies [26], [27]. We implanted a system containing a 64 site array into the mouse hippocampus (Figure 8A). After recovery from surgery, the animal was connected to a flexible 12 wire tethered cable and allowed to explore a 30 cm×50 cm enclosure during acquisition of electrophysiological data. Current source density (CSD) analysis of LFP signals with a vertical resolution of ∼28 µm revealed relatively uniform theta oscillations in layers between the CA1 and dentate gyrus (Figure 8B). However, marked shifts in amplitude and phase were observed on sites around the suprapyramidal and infrapyramidal blades of the dentate gyrus. We also observed theta phase-dependent oscillatory firing of hippocampal neurons recorded from the same device during exploratory behavior (Figure 8C). Neurons were found to be preferentially active in the vicinity of the negative peak of the theta potential, which is consistent with previously reported results [28].

## Discussion

High-density, large-scale electrophysiology has been an elusive goal of systems neuroscience. A prerequisite for implementing this technique is to increase the number of electrodes, and at the same time miniaturize the instrument to make the technology minimally invasive and compatible with small freely moving animals. The electrophysiology system reported here, incorporating a nanofabricated high-density lead probe with an ASIC supporting multiplexed signal transduction, is one of the smallest and lightest fully integrated 64 channel recording instruments developed to date, and the first to include electrode impedance diagnostic and conditioning functionalities. We have demonstrated our system is capable of low noise, wide band spike and LFP measurements in anesthetized and awake behaving mice.

Miniaturization of the interconnecting wires on the probe is an effective method to increase the number and density of recording sites without increasing the device's overall dimensions. Narrower probes interfere less with surrounding neurons, glia, and the blood-brain barrier than larger devices. Moreover, it is speculated that smaller device cross-sections provide favorable conditions for sustaining stable neural interfaces over longer timescales in chronic implants [29], [30]. Further work is underway to determine what, if any, is the maximum cross-section that will avoid inducing excessive acute and chronic damage. It should be noted that the mean spike amplitude of units from the hippocampus of awake mice was less than that of the acute thalamic recordings (∼70 µV vs 150 µV) and appeared to deteriorate over time. We speculate this difference might be attributed to increased neural tissue damage by micromotion of the skull-tethered probe relative to the brain [30], [31]. Thus, in addition to small probe size, attaining high-amplitude units in chronic experiments may require a flexible electrical interconnect between the probe and PCB [32].

The 290 nm leads reported here enabled the fabrication of 64 electrode sites on a single shaft whose width ranged from 60 µm near the tip to 85 µm at the base. This array provided high-density extracellular coverage across 1.3 mm, which is sufficient to span all layers of mouse cortex. Extrapolating from these results, leads would need to be reduced to 58 nm to create a hypothetical 320 site, 85 µm wide probe that spans the entire length of the mouse brain along the dorsoventral axis (∼6.5 mm) with the same density as the array depicted in Figure 2A. The development of such narrow wires will contribute additional noise to the measurement, and presents challenges in fabrication. Capacitive crosstalk is another important consideration in scaling down of lead separation, however, given the sub-100 nm thickness of the wires, parasitic capacitance between adjacent wires is minimal, and can be counteracted by reducing electrode impedance via electroplating [33]. Other approaches for maintaining small device profile, such as integration of multiplexing circuitry directly on the silicon shaft [18], the use of multiple conductor layers, or alternative signal transduction paradigms relying on piezoelectric nanoelectromechanical systems [34], are promising but will require some additional engineering challenges to be resolved before resulting in viable recording devices.

Multichannel arrays output dozens of independent signals, and transferring these data off the recording system for storage and analysis is not trivial. The conventional approach of connecting one external wire per channel would result in a prohibitively large cable bundle tethering the animal to its recording environment. While ultra-fine wire cables with up to ∼100 wires have been used, this approach is not ideal for behavioral studies, and moreover, is unlikely to sustain an additional order of magnitude improvement in the number of recording channels. On the other hand, multiplexed electrophysiology with integrated electronic components is a highly scalable strategy for circumventing the one electrode per cable requirement. Our ASIC multiplexes input signals by a factor of 32, and most ASIC functions can be shared betweeen multiple chips (see Methods). Thus, if a recording system were to be expanded to 128 channels, the cable needed to record data would contain 8 wires (plus 8 additional wires for electroplating/impedance testing). Likewise, readout of an unprecedented 1,024 channels would require 36 wires, corresponding to well over a tenfold reduction in external wiring requirements for electrophysiological recordings. We envision such recordings could be realized via a straightforward extension of the results presented here, by combining nanofabricated silicon probes with multiple 64 channel ASIC chips.

In neurophysiological studies of behavior, there is growing interest in utilizing wireless systems that eliminate the need for cables altogether [35]. However, power requirements invariably force a tradeoff between weight, measurement frequency bandwidth, digital sampling resolution, electronic noise, and number of channels in the wireless transmitter [36]. Current state-of-the-art wide-band, multichannel wireless neural interfaces approach or exceed the size and weight of an adult mouse [37], [38]. Our lightweight wired system affords low noise measurements of spikes and local field potentials, a feature which is absent from some wireless instruments. It is likely that significant advances occur in wireless technology are on the horizon; but until then, wired and multiplexed recording technology will remain particularly attractive for behavioral studies of mice, which are amenable to a broad spectrum of genetically encoded brain circuit dissection techniques [39].

Finally, the wealth of data emerging from high-density multichannel probes presents new challenges and opportunities for data analysis [5], [40]. Significant headway has already been made automating spike classification and sorting routines [41], [42]. Additional work is needed to automatically and reliably extract single-unit spike times from electrode array measurements comprising an arbitrary number of contiguous input channels, and receiving extracellular signals from several co-active neurons.

## Materials and Methods

### Probe nanofabrication

Silicon probe fabrication was carried out on a 100 mm diameter SOI substrate, with a buried oxide (BOX) layer of 1.2 µm, and device layer of 20 µm. The wafer was initially placed in a thermal oxidation furnace at 1000°C to produce a silicon oxide film which was matched in thickness – and hence in stress to the BOX layer. Due to the relatively long time involved in e-beam lithography, we patterned small (≤3 µm) features and the 108 µm^2^ electrode pads with e-beam lithography, and larger features with conventional UV contact lithography. Metal was deposited after each of these steps. Note that the width of the interconnecting leads was only 290 nm on the areas of the array where space was most limited (for example, see Figure 2E). In other areas with more space for routing wires, the width was increased to minimize the likelihood of fabrication-related device defects.

For the e-beam process, a polymethyl-methacrylate (PMMA) resist layer was spin-coated and baked before exposing the nanoscale leads in an EBPG 5000+ writing tool (Leica). Patterning across the wafer containing 170 devices required approximately 7 hours of e-beam writing time on this instrument. After developing the exposed resist, the wafer was transferred to a thermal evaporator for deposition of the first metallic layer (3 nm Cr for adhesion and 70 nm Au). The unpatterned resist and metal were lifted off by immersing the wafer in acetone, briefly applying ultrasonic agitation, and rinsing in isopropyl alcohol. For the second metallization layer involving larger electrically conducting features located at least 400 µm from the narrow front portion of the shaft, a standard contact UV photolithography process was employed. This involved spin-coating a negative tone photoresist (AZ nLOF2020) and baking it onto the wafer, after which the wafer was transferred to mask aligner (MA6, Karl Suss). Patterns belonging to the second metal layer were aligned, with an accuracy of ∼0.8 µm, to alignment marks created during the e-beam lithography step. After resist development, a 3nm Cr/100nm Au layer was thermally evaporated, followed by lift-off in acetone.

Next a 2.2 µm parylene-C layer was coated onto the front side of the wafer; electrode recording sites and wire bond contacts were then exposed by etching the parylene film in O_2_ plasma, using photo-patterned resist as the masking material. The thermal oxide material on the front of the wafer was then similarly masked and etched. Subsequently, the silicon device layer was anisotropically etched via deep reactive ion etching (DRIE, Bosch process), again using the photoresist as masking material. The exposed BOX layer was then etched down to ∼100 nm but not completely removed. The wafer was flipped over and mounted onto a temporary carrier wafer with wax or photoresist. The bottom oxide was completely removed, and XeF_2_, which has high etching selectivity for Si over oxide, was applied to remove the 350–400 µm SOI Si substrate layer. The exposed 100 nm of buried oxide attaching the probes to the remaining SOI frame was then carefully etched with CF_4_ plasma. Finally, the finished probes were released from the temporary carrier with acetone, and rinsed with isopropyl alcohol, ethyl alcohol and deionized water. Devices were created with a shaft length of either 2.5 or 5.5 mm. We found that ∼40% of probes survived the entire process with ≤5 defective channels. The majority (>70%) of defects occurred during the e-beam lithography and subsequent metal liftoff step. We foresee opportunities for improving device yield through further research and refinement of the e-beam lithography process.

### Recording system assembly

Custom-designed PCBs were initially assembled with surface mount resistors and bypass capacitors that perform voltage stabilization and division (5 V to 3 V) for powering and digitally controlling the ASIC. A connector (nano-series, Omnetics) was also soldered onto the PCB. The ASIC was diced to a rectangular shape of 8.2 mm×3.5 mm, and thinned to 250 µm prior to assembly. The neural probe and ASIC were glued onto the PCB with a drop of PMMA dissolved in anisole. A total of 64 wire bonds were then created to connect the probe to the ASIC inputs; an additional 25 bonds were made from the ASIC to the PCB. The electrical contact areas were then encapsulated in opaque epoxy to prevent damage and photoelectric artifacts. Drawings are available from the authors for the PCB and silicon probe designs.

For electrode impedance measurements and gold electrodepostion, the system was connected to a DAQ device (USB-6251, National Instruments) via an intermediate PCB containing circuitry for performing impedance checking and conditioning. The circuitry on this intermediate PCB consisted of mechanical relays for rapidly connecting or disconnecting the reference electrode from ground in order to respectively allow impedance checking or electroplating. In addition, a bank of surface mount R-C components allowed generation of a 1 nA sinusoid from a 1 kHz 5 V square wave, and was used as the bias signal during impedance measurements. Finally, resistors divided a 5 V DC signal to −1.5 V between inputs and reference for electroplating. The ASIC can accommodate a range of other settings, for example, DC potentials of up to ±3 V for electroplating. All inputs to the intermediate PCB were supplied by the same DAQ device used in recording.

After attaining the targeted impedance values, the ground and reference pins of the ASIC were permanently shorted, and a stainless steel ground wire was soldered onto the PCB. For neuronal recordings the system shown in Figure 4 was connected directly to the USB-compatible DAQ device via a flexible 1 meter cable (consisting of 12, 36 gauge wires) plugged into the connector on the PCB. The integrated multiplexers permitted sampling speeds of up to 31.25 kilosamples per second (kSps) per channel. The experiments reported here were carried out at 22 kSps per channel, corresponding to the upper aggregate sampling rate of the DAQ device, which multiplexed signals with 16-bit resolution before sending it to a computer for storage and offline analysis.

The cable provided the following inputs/outputs to the system: (1) 3 V battery-supplied power source; (2) ground, (3 & 4) analog output of the two multiplexers; (5) digital input channel selector, commanding the ASIC to sample the next channel when this line is activated; (6) digital channel indicator (synchronization), which reports a high voltage when the first input channel in the multiplexing sequence is active (i.e., Ch. 1 and 33 for the 1^st^ and 2^nd^ multiplexer, respectively); (7) digital channel reset, which commands the ASIC to return to the first input channel; (8) digital settle, for rapidly discharging all capacitors in the front end amplifiers to ground in the event of amplifier saturation; (9) digital test enable switch, for toggling between recording and impedance test (i.e., check/conditioning) mode; (10 & 11) input voltages for performing impedance tests; and (12) bank selection switch, for toggling between the 1^st^ and 2^nd^ multiplexer during impedance tests. During recording only pins 1 through 6 were used; pins 7 through 12 were either shorted to ground or disconnected from the DAQ device. All 12 pins were connected during impedance measurements, although pin 8 was never activated. The system was operated with software created in LabVIEW (National Instruments), and data was analyzed offline with Matlab (MathWorks). To convert amplified signals to input-referred voltage we divided them by 200, which corresponds to the nominal gain of the amplifiers (actual measured gain  = 194). Note that most ASIC functions (pins 1, 2, 5–9, 12) can be shared across multiple chips to maintain low wiring requirements for even greater numbers of channels.

### Electrode plating

The probe was immersed in gold plating solution (Sifco), and a −1.5 V DC potential was applied through the 11^th^ control terminal on the active channel relative to a platinum wire reference. We used mechanical relays on the intermediate PCB described above to rapidly alternate between electroplate and impedance check modalities with the ASIC. Channels were selected by controlling the 5^th^ and 12^th^ ASIC terminals described above. For electrophysiological recordings, electrodes were plated until their impedance fell into the range of 0.4 to 0.8 MΩ unless stated otherwise in the main text. If the impedance would not drop below 3 MΩ, that recording site was classified as faulty and excluded from further analysis. To minimize the likelihood of short-circuiting adjacent recording sites by excessive electroplating, the impedance was not lowered below 80 kΩ.

We observed a small increase in the impedance of electroplated channels after an in vivo experiment, which was likely due to detachment of a portion of the electrodeposited gold material during insertion. For example, after one acute experiment the probe's impedances rose from 0.5±0.3 MΩ to 0.8±0.4 MΩ, corresponding to an increase of 0.3±0.3 MΩ per channel (values denote mean±s.d.).

### Electrode impedance measurements

Impedance was measured by feeding a 1 nA, 1 kHz sinusoidal current into the ASIC, and reading out resulting the voltage signal from the multiplexer. Impedance was calculated as the ratio of AC voltage to current. Values were confirmed by comparing measurements to a commercial impedance measurement device (nanoZ, White Matter LLC). To obtain good correspondence with the nanoZ, a correction factor of 0.62 had to be applied to the V/I ratio as measured by the ASIC and accompanying instrumentation. All impedance measurements reported here were made in phosphate buffered saline (PBS).

### Noise measurements

The probe was immersed in PBS solution, and 200 s duration multiplexed measurements were carried out on 64 channels with ∼22 kHz sampling rate per channel (exact value: 1/44.8 µs for all experiments). Root-mean square noise values were obtained by taking the standard deviation of each channel's voltage-time trace after filtering from 0.1–6.5 kHz with a 3^rd^ order Butterworth filter. No additional signal conditioning, e.g., notch filter was applied. The noise spectral density (NSD) was obtained from the raw demultiplexed data using the Welch periodogram estimation method.

### Amplifier gain and phase measurements

The nominal gain and phase response of the amplifier was measured by applying sinusoidal voltage signals from a benchtop signal generator (DS345, Stanford Research Systems) and measuring the amplifier output signals on an oscilloscope (Figures S1A,B). Filter-related phase and gain changes were observed at frequencies near the bandpass filter cutoff frequencies, which correspond to 1.3±0.2 and 6,400±300 Hz, respectively. These responses are a normal property of filters. Note that the use of off-chip passive components would allow even wider band recordings (as low as 0.02 Hz and high as 20 kHz with this ASIC), but we opted not to implement this option in these experiments in favor of minimizing system size.

In order to accurately interpret LFP phase information, we first confirmed that the ASIC's analog high-pass filter and any electrode impedance-dependent distortions in the phase and amplitude of electrophysiological signals were minimal [43]. To calibrate the phase shift properties of our probes as measured by the ASIC, we took measurements with the same probe used in generating data for Figure 5. A single-tone sinusoidal stimulus with specified frequency was applied in a PBS bath. We monitored the relative phase and amplitude between the reference signal, and the signals transduced by the 64 channel system (Figure S1C). The probe contained functional sites with an impedance range of 0.23 to 3.16 MΩ, spanning the full range of plausible impedance values in our recordings. We found a phase dispersion at f = 1.1 Hz of 1.1° (s.d.; full range is 7.3°; Figure S1D), and gain dispersion of 3% (s.d.; full range is 18%; Figure S1E), confirming that variations in electrode impedance will not interfere with LFP phase analysis. The gain dispersion provided similarly tight tolerances for the majority of input channels; however, an 18% variation found for one of the inputs may present an unacceptable source of error in some applications, such as CSD analysis. To ensure that CSD analysis was not adversely affected by such electrodes, we performed amplifier gain measurements with freshly gold-plated electrodes prior to surgery, and excluded such channels from CSD calculations.

### Electrophysiology in mice

All surgical procedures were approved by the Institutional Animal Care and Use Committee at the California Institute of Technology (NIH Assurance: A3426-01). For acute anesthetized recordings, 25–30 g male C57BL/6 mice ordered from Jackson Labs were anesthetized with chloral hydrate (400 mg/kg, i.p.), and placed in a stereotaxic frame apparatus (Kopf Instruments). Body temperature was regulated with a heating pad. The depth of anesthesia was regularly checked by pinching the tail and toe of the animal, and additional anesthetic dose was given as necessary. After reflecting skin on the scalp, a ∼0.5 mm^2^ rectangular craniotomy was made with a drill (−2.00 mm AP and 1.50 mm ML relative to bregma), taking care to not rupture the dura. A slit was made in the dura with forceps, and the dura was frequently moistened with saline. The recording system was mounted onto a motorized micromanipulator (Sutter MP-225), and the probe lowered into the brain at ≤10 µm/s, pausing frequently to record spontaneous activity. Probes used in acute experiments were reused by cleaning in trypsin (Invitrogen) for 20 min. and rinsing with DI water, measuring impedance, and re-plating electrodes if the mean impedance across all functional sites exceeded 1 MΩ. Re-plating was usually required after 3 or 4 experiments.

For electrophysiology in awake behaving mice, animals were anesthetized with isoflurane, and body temperature regulated with a heating pad. Ketoprofen was administered intramuscularly (1 mg/kg) to prevent swelling. Sterilized equipment was used throughout the surgical procedure. After cleaning and exposing the skull, a stainless steel reference electrode wire was inserted into the cerebellum. Two stainless steel mechanical anchor screws were fixed onto the anterior and posterior edges of the exposed skull by means of dental cement (C&B Metabond, Parkell Inc.). A rectangular craniotomy for the probe was made at −1.90 mm AP and −1.4 to −1.8 mm ML relative to bregma. The probe tip was lowered to a final depth of 2 mm relative to bregma. The craniotomy was then sealed with a biocompatible silicone elastomer (Kwik Kast, WPI Inc.), followed by dental cement to secure the printed circuit board to the bone screws. The printed circuit board reference wire was then soldered to the animal reference wire, and covered with more cement. The animal was allowed to recover in an isolated, heated cage and monitored daily. Analgesic and antibiotic medication was administered in the drinking water for one week post-operatively. Electrophysiological recording sessions lasting 1 hour began as early as 1 day post-op in fully recovered and alert animals, and continued almost daily for 10 days post-operatively. To verify the location of the devices, animals were deeply anesthetized with ketamine/xylazine and transcardially perfused. 75 µm thick brain sections were stained with hematoxylin (Vector Laboratories), washed with DI water, and mounted on glass slides. Probe tracks appeared as two small parallel scars when viewed under a bright field microscope.

### Electrophysiological signal analysis

Offline spike analysis consisted of first demultiplexing raw data to separate the multiplexed signal into its constitutive input channels. Signals were subsequently bandpass filtered from 0.4–5 kHz with a 3^rd^ order Butterworth filter. In the case of measurements in freely behaving animals, motion artifacts were removed by subtracting the mean instantaneous signal of all functional channels in the array. Spikes were detected based on a negative amplitude threshold method (threshold≈10 times the 68^th^ percentile range, corresponding to ∼40 µV). Isolation of waveforms into putative single units was carried out using a multichannel template matching process. Spike sorting began by establishing the waveform templates for each putative unit based on Euclidean distance comparisons of ∼1 ms waveform segments. Finally, spikes were assigned to a particular template if the distance to that template was less than to other templates, and if the distance did not exceed a preset threshold. This approach appeared to provide single-unit discrimination for non-overlapping spike events; additional work is needed to analyze simultaneously firing neurons observed on the same set of channels [41], [44].

Current source density analysis was carried out with the CSDPlotter program developed for Matlab [45] (iCSD method), using an electrode vertical separation of 0.0275 mm (approximate separation for the array illustrated in Figures 2B and 3B), and standard deviation of 0.055 mm. One of the 32 recording channels on the shaft were excluded because it appeared to be defective. Signals used for the CSD plot were filtered from 3–13 Hz with a 1^st^ order Butterworth filter.

For spike-phase dependence analysis, demultiplexed signals were filtered in the theta band with a 4^th^ order Chebyshev filter from 6–10 Hz and decimated by a factor of 20. Phase was determined by calculating the Hilbert transform of the data and then calculating the arctangent of the real and imaginary components.

## Supporting Information

Figure S1 **Low gain and phase dispersion of the ASIC amplifiers.** (**A**) Gain and phase response measurements of an amplifier on the ASIC. (**B**) Same as (A), but the plot range has been reduced. (**C**) Illustration depicting phase offset between a 1.1 Hz reference signal (in this case, from a function generator) and the signal measured through an electrode on the silicon probe and amplifier. Signal amplitude is normalized for illustration purposes. (**D & E**) Phase offset and gain versus frequency. Dashed lines and arrows denote the full measured range of dispersions at 1.1 Hz. The standard deviation of phase and gain dispersion for LFP frequencies (≥1 Hz) is 1.1° and 3%, respectively. Blue dots: response of 31 individual input channels with 1 kHz impedance of 0.23–3.16 MΩ.
(TIF)

Table S1 **Comparison of number of recording channels, device size, and channel density for a representative subset of neural probes described in the literature.** The table cites literature describing planar implantable microelectrode arrays similar to the one in this paper. Large channel-to-width ratios imply minimally invasive shafts containing high-density recording arrays. Due to the use of nanofabrication techniques, the probes presented in this publication offer among the highest channel-to-width ratios and areal recording site density reported to date. **Notes:** (a) 3D microassembly; (b) contains integrated electronics, and only 8 of 188 channels can be read out at any given time; (c) assume 9 channels per shaft and 500 µm shaft spacing; (d) assume 8 channels per shaft and 400 µm shaft spacing.
(DOCX)
