# Supplementary figures and images for: Multiplexed, High Density Electrophysiology with Nanofabricated Neural Probes
Source: PLoS One. 2011 Oct 12;6(10):e26204. doi: 10.1371/journal.pone.0026204 (PMC3192171; doi:10.1371/journal.pone.0026204)

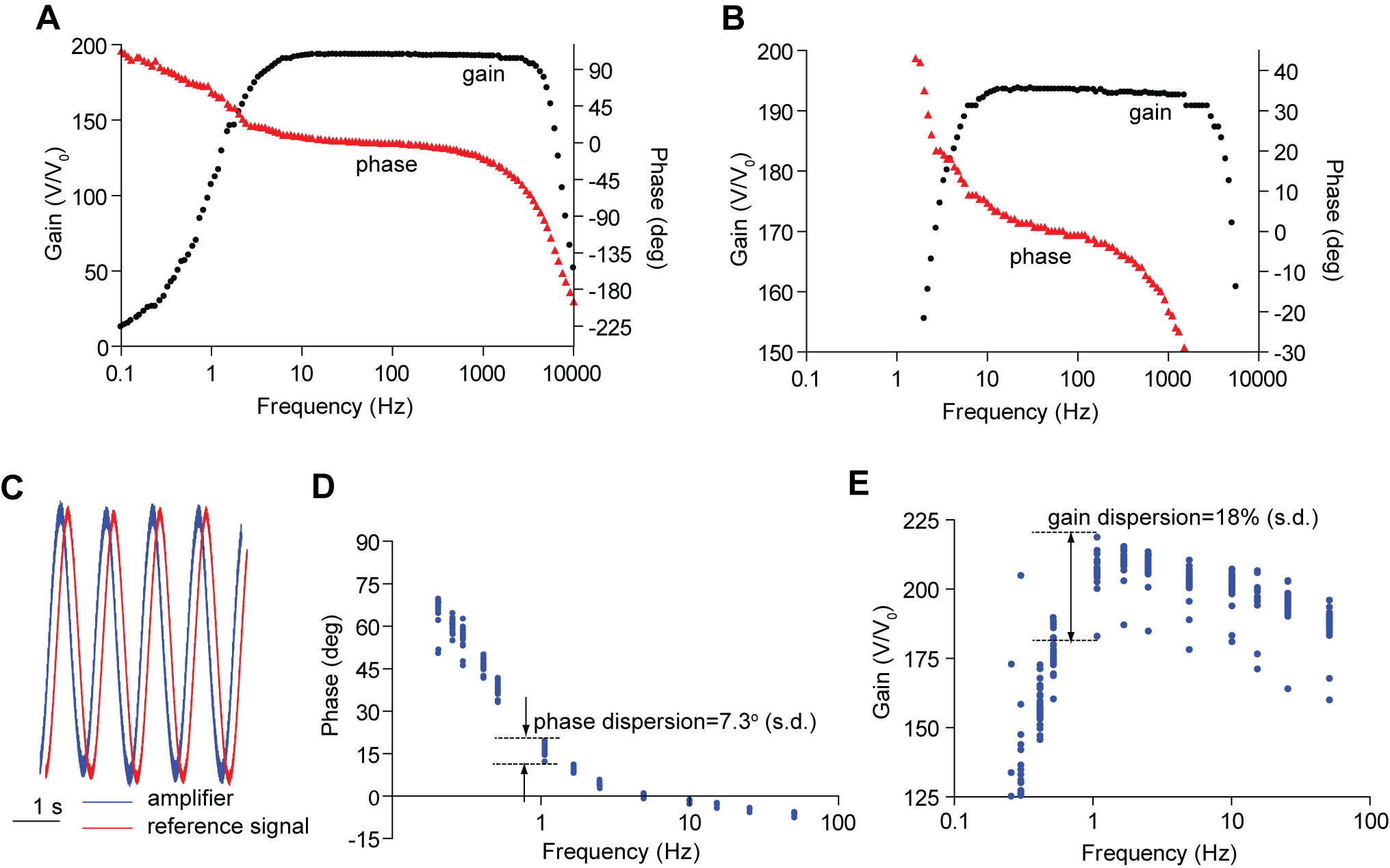

Supplement: Figure S1 — Low gain and phase dispersion of the ASIC amplifiers. (A) Gain and phase response measurements of an amplifier on the ASIC. (B) Same as (A), but the plot range has been reduced. (C) Illustration depicting phase offset between a 1.1 Hz reference signal (in this case, from a function generator) and the signal measured through an electrode on the silicon probe and amplifier. Signal amplitude is normalized for illustration purposes. (D & E) Phase offset and gain versus frequency. Dashed lines and arrows denote the full measured range of dispersions at 1.1 Hz. The standard deviation of phase and gain dispersion for LFP frequencies (≥1 Hz) is 1.1° and 3%, respectively. Blue dots: response of 31 individual input channels with 1 kHz impedance of 0.23–3.16 MΩ. (TIF) [file pone.0026204.s001.tif]
