# Supplementary material for: Multiplexed, High Density Electrophysiology with Nanofabricated Neural Probes
Source: PLoS One. 2011 Oct 12;6(10):e26204. doi: 10.1371/journal.pone.0026204 (PMC3192171; doi:10.1371/journal.pone.0026204)
Supplement: Table S1 — Comparison of number of recording channels, device size, and channel density for a representative subset of neural probes described in the literature. The table cites literature describing planar implantable microelectrode arrays similar to the one in this paper. Large channel-to-width ratios imply minimally invasive shafts containing high-density recording arrays. Due to the use of nanofabrication techniques, the probes presented in this publication offer among the highest channel-to-width ratios and areal recording site density reported to date. Notes: (a) 3D microassembly; (b) contains integrated electronics, and only 8 of 188 channels can be read out at any given time; (c) assume 9 channels per shaft and 500 µm shaft spacing; (d) assume 8 channels per shaft and 400 µm shaft spacing. (DOCX) [file pone.0026204.s002.docx]

**Supporting Table:**

| **Publication source** | **# channels per shaft** | **Shaft width at base (μm)** | **Channels-to-width ratio (per shaft)** | **Electrode areal density (# channels per mm^2^)** | **Electrode volumetric density (# channels per mm^3^)** |
| --- | --- | --- | --- | --- | --- |
| this paper, Figure 1A | 64 | 85 | 0.75 | 49 | 49 |
| this paper, Figure 1B | 32 | 60 | 0.53 | 64 | 64 |
| this paper, Figure 1C | 16 | 40 | 0.40 | 64 | 64 |
| [[46](#_ENREF_46)] | 8 | 61 | 0.13 | 24 | 96^a^ |
| [[18](#_ENREF_18)] | 188 | 180 | 1.04 | 47 | 47^b^ |
| [[47](#_ENREF_47)] | 9 | 140 | 0.06 | 27 | 81^a,c^ |
| [[48](#_ENREF_48)] | 16 | 220 | 0.07 | 16 | 16 |
| [[49](#_ENREF_49)] | 16 | 70 | 0.23 | 32 | 32 |
| [[50](#_ENREF_50)] | 8 | 50 | 0.16 | 24 | 96^a,d^ |
| [[11](#_ENREF_11)] | 54 | 212 | 0.25 | 54 | 54 |
| [[51](#_ENREF_51)] | 8 | 50 | 0.16 | 48 | 48 |
| [[51](#_ENREF_51)] | 16 | 85 | 0.19 | 64 | 64 |
| [[21](#_ENREF_21)] | 4 | 80 | 0.05 | 16 | 16 |
| [[15](#_ENREF_15)] | 8 | 25 | 0.32 | 32 | 32 |

**Table S1. Comparison of number of recording channels, device size, and channel density for a representative subset of neural probes described in the literature.** The table cites literature describing planar implantable microelectrode arrays similar to the one in this paper. Large channel-to-width ratios imply minimally invasive shafts containing high-density recording arrays. Due to the use of nanofabrication techniques, the probes presented in this publication offer among the highest channel-to-width ratios and areal recording site density reported to date. **Notes:** (a) 3D microassembly; (b) contains integrated electronics, and only 8 of 188 channels can be read out at any given time; (c) assume 9 channels per shaft and 500 μm shaft spacing; (d) assume 8 channels per shaft and 400 μm shaft spacing.
